# Challenging underwater endoscopic mucosal resection of duodenal adenoma successfully achieved with combination of therapeutic pediatric colonoscope and retroflexion

**DOI:** 10.1055/a-2474-6919

**Published:** 2024-12-10

**Authors:** Amina Abdulle, Mathieu Pioche, Melissa Gruner, Alexandru Lupu, Gwladys Pointet, Pierre Lafeuille, Jérôme Rivory

**Affiliations:** 1 36609 Department of Gastroenterology and Endoscopy, Edouard Herriot Hospital, Lyon, France; 2 9314 Department of Medical Sciences, University of Turin, Turin, Italy; 3 423788 Department of Gastroenterology and Endoscopy, Croix Rousse Hospital, Lyon, France

Nonampullary duodenal adenomas are rare, detected in only 0.1%–0.4% of patients undergoing esophagogastroduodenoscopy (EGD) 1 . The European Society of Gastrointestinal Endoscopy (ESGE) recommends that all duodenal adenomas should be considered for endoscopic resection, as progression to invasive carcinoma is highly likely 2 . Endoscopic resection has been a first choice of treatment for duodenal adenomas, but the method has not been standardized 3 . To overcome the practical difficulty of conventional endoscopic mucosal resection (EMR), underwater endoscopic mucosal resection (UEMR) has recently been developed. UEMR seems to have significantly higher R0 resection rates and lower post-procedural bleeding rates than EMR. Moreover, it seems safer than conventional EMR and has been associated with a lower incidence of recurrences 4 5 .

We report a case of a 63-year-old patient hospitalized after multiple episodes of biliary cholangitis. The endoscopic retrograde cholangiopancreatography (ERCP) procedure showed the presence of a potentially adenomatous lesion located proximal to the papilla ( Fig. 1 ). Biopsies confirmed high grade dysplasia.

**Fig. 1:**
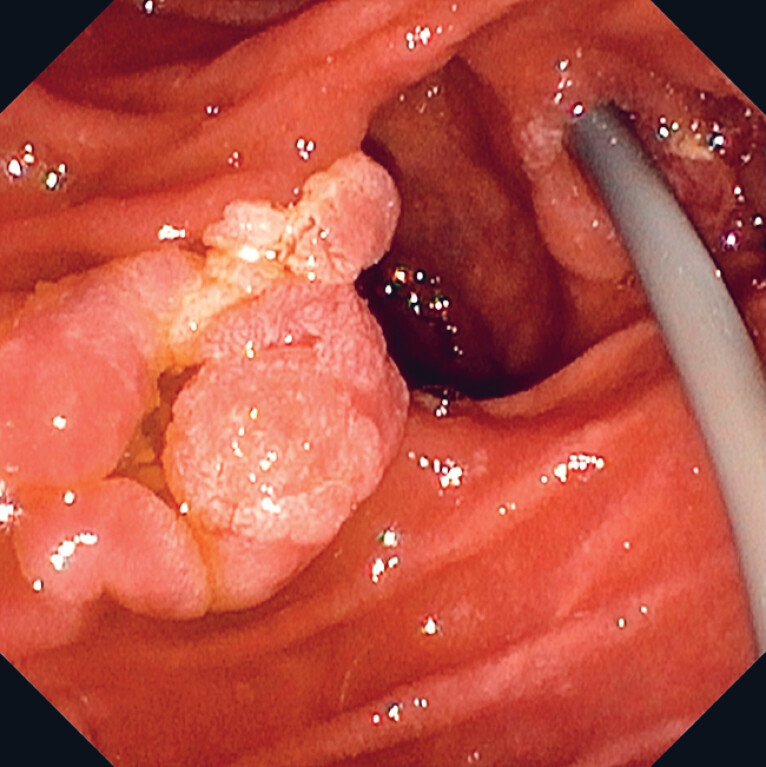
Duodenal adenoma partially involving a periampullary diverticulum.

The adenoma was first approached using a duodenoscope. The lesion measured approximately 20 to 25 mm and was partially located inside a diverticulum. A piecemeal cold snare mucosectomy was performed, combining 15- and 10-mm snares. To reach a remnant part of the lesion impossible to access with the duodenoscope, we used a pediatric therapeutic colonoscope to achieve the resection. The mucosectomy was completed by a combination of different strategies: the underwater technique allowed keeping the remnant adenoma floating, but stability was obtained thanks to a retroflexed position of the scope in the second duodenum. Finally, we obtained a macroscopically complete resection ( Fig. 2 , Video 1 ). No immediate or delayed adverse events occurred and histological examination revealed high grade dysplasia. A 4-month follow-up showed no recurrence.

**Fig. 2:**
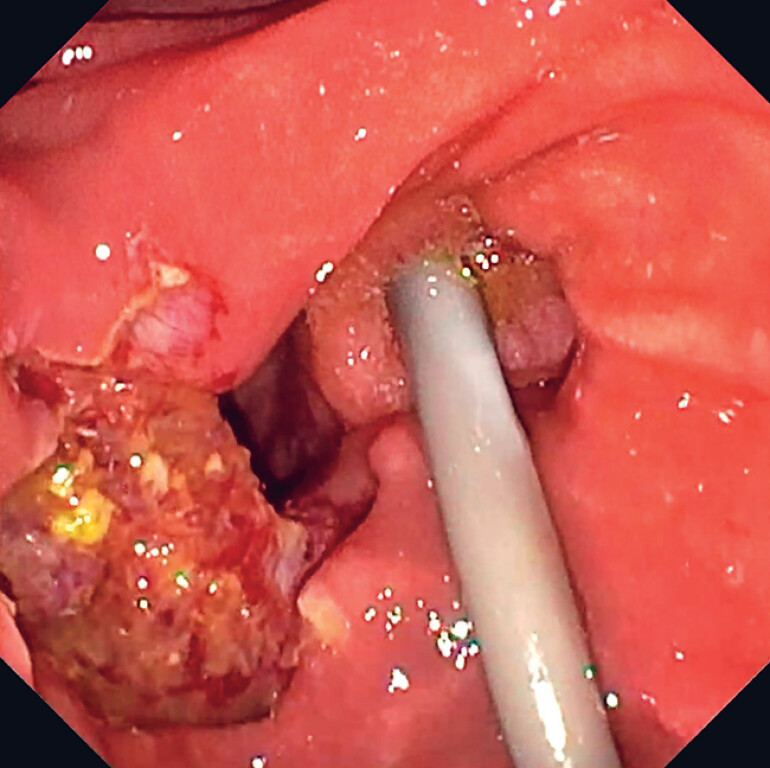
Macroscopically complete resection of the adenoma.

Challenging resection of a duodenal adenoma successfully achieved by combining the use of underwater endoscopic mucosal resection, a therapeutic pediatric colonoscope, and retroflexion.Video 1

An underwater strategy combined with retroflexion can be a way to obtain complete endoscopic resection when access to a duodenal adenoma is hampered due to its periampullary diverticular location.

Endoscopy_UCTN_Code_TTT_1AO_2AG_3AC

## Notes

Endoscopy E-Videos https://eref.thieme.de/e-videos
*E-Videos* is an open access online section of the journal *Endoscopy* , reporting on interesting cases and new techniques in gastroenterological endoscopy. All papers include a high-quality video and are published with a Creative Commons CC-BY license. Endoscopy E-Videos qualify for HINARI discounts and waivers and eligibility is automatically checked during the submission process. We grant 100% waivers to articles whose corresponding authors are based in Group A countries and 50% waivers to those who are based in Group B countries as classified by Research4Life (see: https://www.research4life.org/access/eligibility/ ). This section has its own submission website at https://mc.manuscriptcentral.com/e-videos .
